# Identification and analysis of HML2 sequences in human genome assembly GRCh37/hg19

**DOI:** 10.1186/1742-4690-10-S1-P9

**Published:** 2013-09-19

**Authors:** Marta Cadeddu, Laura Vargiu, Patricia Rodriguez-Tomé, Göran O Sperber, Jonas Blomberg, Enzo Tramontano

**Affiliations:** 1 Dept. of Life and Environmental Sciences, Cagliari, Italy; 2 Center for Advanced Studies, Research and Development, Sardinia, Italy; 3 Physiology Unit, Dept of Neuroscience, Uppsala, Sweden; 4 Section of Virology, Dept. of Medical Sciences, Uppsala, Sweden

## Background

Human endogenous retroviruses (HERVs) originated from exogenous retroviral infections of the human germ line cells and spread in the human population through vertical transmission over millions of years. Among HERVs, the HML2 proviruses [1] are the most recently integrated and show the most intact proviral genomes. HML-2 expression has tentatively been associated with different pathological conditions, including Hodgkin’s lymphoma, melanoma, breast and testicular cancer. A comprehensive recent study identified 91 HML2 proviruses [2].

## Material and methods

Human genome (assembly GRCh 37/hg19) was analyzed with RetroTector (ReTe) version 1.01 [3]. ReTe was run on a machine with 4 6-core Xeon processors, 2.66Ghz each, 256 Gb of RAM and 4 Tb of disks, with an estimated execution time of 1-2 days. BLASTN, using HML consensuses (Blikstad *et al*, unpublished) and the May 2013 Repeatmasker library, ENSEMBL and MEGA5 were used, in successive steps, for classification and identification of locus position and phylogenetic inference. Time since integration was inferred using a neutral substitution rate between cognate LTRs of 0.2 mutations per million years.

## Results

ReTe [2] identified more than 120 HML2 proviruses, many of which were not previously reported, accounting for roughly 0.01% of the total human genome. Among the identified HML2 proviruses more than 50% are ≥ 8000 bp in length and more than 50% have both LTRs. HML2 proviruses bordering to HML1, HML3, HML9 and HML10, as well as recombinant proviruses containing HML2 sequences were detected. HML2 proviruses were present in all chromosomes and showed to form clusters, particularly in chromosomes 1, 4, 8 and 19. Open reading frames (ORFs) predicted by ReTe revealed that 21 proviruses have at least 1 ORFs in *gag*, *pro*, *pol* and *env* genes, while 6 had ORFs in 3 genes. Age analysis versus reductions of ORFs and proviral length was performed. Phylogenetic analyses were performed with whole element DNA, concatenated Gag, Pro and Pol amino acid sequences, and Pol amino acid sequences.

## Conclusions

In an attempt to establish a comprehensive catalog of HML2 proviruses that could set the basis for further research, we detected over 120 HML2 proviruses and performed a first characterization of them.
